# Diagnosis by the Urine, or the Practical Examination of Urine, with Special Reference to Diagnosis

**Published:** 1899-07

**Authors:** 


DIAGNOSIS BY THE URINE, or the Practical Examination of Urine, with Special Reference to Diagnosis. By ALLARD MENNINGER, M. D., Professor of Chemistry, Urinology and Hygiene in the Medical College of the State of South Carolina; Visiting Physician to the City Hospital of Charleston, etc., etc. Second edition, enlarged and revised. With illustrations. Philadelphia: P. Blakiston's Son & Co. 1899.

The exhaustion of the first edition, together with the advance in science and knowledge gained by the author since the publication of the first edition, has rendered a thorough review necessary. This has been carefully done, especially in the articles relating to tube casts and the significance of their presence, also in the chapter on the differential diagnosis of Bright's disease, as based on a classification of the normal absolute, the absolute and the relative absolute of solids and urea.

Thus improved and enlarged the work is to be commended to those who desire brevity and conciseness. The author is methodical in his treatment of the different chapters and his efforts in this new edition are to be appreciated. W. C. K.

